# Cystine and theanine: amino acids as oral immunomodulative nutrients

**DOI:** 10.1186/2193-1801-2-635

**Published:** 2013-11-26

**Authors:** Shigekazu Kurihara, Tetsuro Shibakusa, Kenji AK Tanaka

**Affiliations:** Institute for Innovation, Ajinomoto Co, Inc, 1-1 Suzuki-cho, Kawasaki-ku, Kawasaki-shi, Kanagawa, 210-8681 Japan

**Keywords:** Cystine, Theanine, Glutathione, Vaccine, Exercise, Surgery

## Abstract

The decreases in the glutathione (GSH) level in the mouse spleen and liver after immune stimulation are suppressed by the oral administration of cystine and theanine (CT). GSH is considered to be important for the control of immune responses. Antibody production in mice after infection is enhanced by the oral administration of CT. In humans, also, the oral administration of CT has been confirmed to enhance antibody production after vaccination against Flu and also reduce the incidence of cold. However, the GSH level is reduced by intense exercise and surgery. In clinical studies of body-builders and long-distance runners, the intake of CT suppressed excessive inflammatory reactions and a decline in immune functions after intense training. Surgery as well as intense exercise induces excessive inflammatory reactions. In mice, the preoperative administration of CT suppressed excessive inflammatory reactions associated with surgery and promoted the postoperative recovery. Moreover, in clinical studies of gastrectomized patients, CT intake suppressed excessive postoperative inflammatory reactions and induced early recovery. If infection is regarded as invasive stress, CT intake is considered to exhibit an immunomodulatory effect by suppressing the decrease in GSH due to invasive stress. The clarification of their detailed action mechanisms and their application as medical or function foods is anticipated.

## Introduction

Cystine is a sulfur-containing amino acid consisting of 2 cysteine molecules connected by an S-S bond. This sulfur-containing amino acid is one of the precursors of glutathione (GSH), which is vital for antioxidant reactions in the body, and its supply is considered to be a rate-limiting factor in GSH synthesis (Grimble 2006;Rimaniol et al. 2001). GSH has been shown to play an important role in the regulation of immune functions as well as in antioxidant reactions in the body, and is known to decrease when the body is exposed to stress such as intense exercise and surgery (Droge and Holm 1997;Luo et al. 1996;Margonis et al. 2007). Theanine (γ-glutamylethylamide) is an amino acid abundant in green tea and is known to be absorbed after oral ingestion through the small intestine and hydrolyzed into glutamate and ethylamine in the intestine and liver (Asatoor 1966;Bukowski et al. 1999). Indeed, the blood level of glutamate was reported to significantly increase after the intake of green tea or a capsule containing theanine (Scheid et al. 2012).

**Figure 1:**
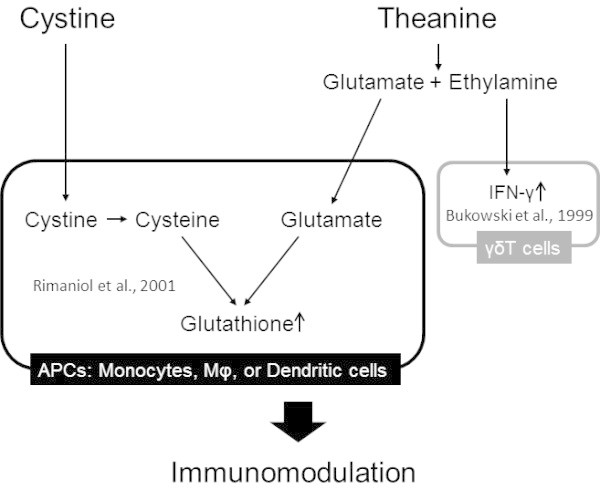
**Working hypothesis regarding the immunomodulative actions of cystine and theanine.** After the oral administration of cystine and theanine, cystine is incorporated into antigen-presenting cells (APCs: monocytes, Mϕ, or dendritic cells) which express cystine transporter (xCT/4F2hc) and reduced to cysteine. Theanine is hydrolyzed into glutamate and ethylamine and the glutamate is incorporated into APCs. The incorporated cysteine and glutamate enhance glutathione synthesis (Rimaniol et al. 2001) and then induce immunomodulative activity. On the other hand, the ethylamine derived from theanine acts on γδT cells (Bukowski et al. 1999).

An experiment using human peripheral blood macrophages (Mϕ) showed that the intracellular GSH content was dose-dependently increased by treatment of Mϕ with cystine, and was increased further by the addition of glutamate (Rimaniol et al. 2001). This report suggests that the GSH content is increased additively or synergistically by the simultaneous intake of cystine and glutamate. However, most of the glutamate orally ingested is known to be metabolized in the small intestine and not to enter the circulation (Windmueller and Spaeth 1975). As mentioned above, theanine, a glutamate derivative, is metabolized into glutamate and ethylamine in the intestine and liver after oral intake. Therefore, theanine is considered to function as a donor that supplies glutamate to the body (Figure 1). In fact, in an experiment using mice orally administered cystine and theanine alone or in combination before immune stimulation, no significant increase in the GSH level was observed in the liver after immune stimulation in the groups administered either agent alone compared with the control group, but it was significantly increased, and the antigen-specific immunoglobulin (Ig) G production in blood was also significantly augmented, in the group administered both agents (Kurihara et al. 2007). Furthermore, in an experiment using influenza-infected old mice, a combination of cystine and theanine (CT) was confirmed to increase GSH synthesis and enhance resistance to infection (Takagi et al. 2010). From these results of animal experiments, not yet confirmed in human trials, the oral administration of CT is considered to increase GSH synthesis and reinforce immune functions. We, therefore, performed studies using several models to clarify the usefulness of CT for strengthening immune functions in humans. These studies are summarized and reviewed.

### Immune response-improving effect after vaccination against influenza in older people

Vaccination against influenza is important in older people for preventing exacerbation of the course of, and reducing the mortality due to, the disease (Jefferson et al. 2005;Mullooly et al. 1994;Nordin et al. 2001). However, as immune functions decline with aging, enhancement of the effectiveness of vaccination is considered important in older people (McElhaney et al. 1990;Remarque et al. 1996;Vu et al. 2002). Therefore, a clinical study was performed to confirm the effectiveness of CT intake at vaccination against influenza in 67 users of a nursing home affiliated to a hospital (mean age: 77 years) (Miyagawa et al. 2008). The 67 institutionalized elderly people, who were given sufficient explanation about the clinical study and consented to participate in it, were randomly divided into placebo and CT groups, and given either a placebo or CT once a day for 2 weeks prior to vaccination. The effectiveness of vaccination was analyzed by examining the antibody level (HI titer) in blood 1 month after vaccination. The rate of seroconversion, which is the percentage of people who have acquired an antibody level effective for the prevention of infection by vaccination among those who did not show an effective antibody level before vaccination, was higher in the CT group than in the placebo group regardless of the vaccine type (type A (H1N1), type A (H3N2), or type B), but the differences were not significant (Miyagawa et al. 2008). Therefore, we performed analysis by stratification using nutritional parameters in blood reportedly related to an aging-associated decline in the immune function (Hara et al. 2005), and observed a marked increase in the rate of seroconversion, particularly for type A (H1N1), in the groups with subaverage blood total protein and hemoglobin levels, i.e., those in a relatively poor nutritional state (Miyagawa et al. 2008). These results suggest that CT enhances the effectiveness of vaccination against influenza in older people with aging-associated declines in immune functions, and that this effect is more notable in older people with a poorer nutritional state. However, more trials in older people are needed, especially in those with malnutrition, to ascertain the effectiveness of CT intake after vaccination against influenza in the elderly.

### Cold-preventing effect

The common cold is an infection usually caused by the entry of viruses into the upper airway in the dry season (Heikkinen and Jarvinen 2003). This process of infection can be naturally prevented more often if the host’s immune competence increases. We performed a clinical study to evaluate the effect of CT intake on vulnerability to the common cold in winter in 176 adult male volunteers (mean age: 40 years) (Kurihara et al. 2010). The 176 adult males, who were given an explanation about the clinical study and consented to participate in it, were randomly divided into placebo and CT groups, administered the assigned preparation daily for 5 weeks, and answered a questionnaire concerning the body temperature and various common cold symptoms daily. The responses to the questionnaire were quantified according to the literature on clinical studies of cold and influenza infection (Hayden et al. 1999;Hayden et al. 1997), conditions of the appearance of various symptoms of cold and the occurrence of cold itself were defined using our original method, and the records during the 5-week period were analyzed. In the CT group, the frequency of fever and chills, which are cold symptoms, was significantly lower, and symptoms due to inflammation (nasal secretion, throat pain, etc.) were milder, compared with the placebo group. Moreover, the incidence of cold and total number of days with symptoms during the investigation period were significantly reduced (Kurihara et al. 2010). From these results, CT intake (980 mg/day, containing 700 mg of cystine and 280 mg of theanine) is considered to be effective for the prevention of cold and its various symptoms due to inflammation during winter in middle-aged and elderly people. Future human trials may reveal the specific effects of CT intake on cold prevention.

### Suppressive effect against decline in immune functions due to intense exercise

It is known that we become more vulnerable to infections including cold after vigorous exercise such as marathon running (Nieman 1997). Vigorous exercise induces excessive inflammatory reactions, promotes the secretion of stress hormones such as corticosterone and glucagon, and weakens the immune functions of individuals (Gleeson and Pyne 2000;Suzuki et al. 2000). Moreover, disruption of the immune system associated with vigorous exercise has been reported to lead to problems during training and the poor physical condition of athletes, such as in overtraining syndrome (MacKinnon 2000;Smith 2003). We, therefore, carried out clinical studies to evaluate the effects of CT intake on changes in immune functions before and after intense training using college body-builders as a model of resistance training and college long-distance runners as a model of endurance exercise (Kawada et al. 2010;Murakami et al. 2009). First, 15 male body-builders who provided informed consent to this clinical study were randomized to placebo and CT groups and given the test food once a day at dinner during a 2-week training period. The training intensity was doubled during the final week compared with the first week of the training period, and changes in blood natural killer (NK) cell activity were serially monitored. In the placebo group, NK cell activity decreased gradually after the beginning of training compared with the pre-training level, with a significant drop after 2 weeks. In the CT group, on the other hand, no decrease in NK cell activity associated with intense training was noted (Kawada et al. 2010). Similarly, 15 male college long-distance runners who consented were randomized to placebo and CT groups and given the test food once a day at dinner for 10 days during a regular training period. Thereafter, they participated in an 11-day summer training camp, and changes in immunological parameters in blood before and after this period were analyzed. In the placebo group, the neutrophil count and high-sensitivity CRP level, which are blood inflammation markers, increased significantly, and the number of lymphocytes, which are immunocompetent cells in blood, decreased after the camp, but no significant change in these parameters was noted in the CT group (Murakami et al. 2009). These results suggest that CT suppresses an increase in inflammatory reactions associated with the stress of sustained intense exercise such as that during a training camp, and prevents an associated decline in immune functions. Therefore, we further analyzed changes in blood immunological parameters immediately after intense endurance exercise in the long-distance runners. Similarly, 16 male college long-distance runners who consented were randomized to placebo and CT groups and given the test food once a day at dinner for 7 days of regular training and 9 days during a training camp (daily for a total of 16 days). Interval training of 1,000 m × 15 times was performed as intense endurance training before breakfast on the first and last days of the camp, blood was sampled before and after the training, and changes in blood markers were analyzed. The neutrophil count and myoglobin level showed marked increases, but the lymphocyte count decreased, due to intense exercise stress on the first day of the camp, but these changes were significantly milder in the CT group than in the placebo group (Murakami et al. 2010). On the last day of the camp, the changes in blood markers associated with intense exercise were reduced, probably due to the physical and mental effects of the training, and no particular effect of CT intake was noted at this point. The results on the last day suggest that CT exert no effect when inflammatory reactions due to exercise stress are mild. Thus, CT are considered to suppress excessive inflammatory reactions without inhibiting physiologic and necessary inflammatory reactions. From the results of these clinical studies involving athletes, CT are considered to suppress excessive inflammatory reactions induced by severe stress, such as that due to intense exercise training, and prevent a decline in immune functions.

### Promotion of recovery after surgery

#### Postoperative anti-inflammatory/recovery-promoting effects in mouse surgery models

Surgery was selected as an invasive stress other than exercise. Generally, laparotomy, which involves more intestinal manipulation, is considered to cause severer inflammation than endoscopic surgery (Hiki et al. 2006). Therefore, using a mouse intestinal manipulation model (Kalff et al. 1998) as a model of laparotomy, the effects of the preoperative oral administration of CT on inflammation associated with surgical stress were evaluated according to changes in the blood inflammation marker interleukin (IL)-6 level (Biffl et al. 1996). Compared with a sham operation group, the blood IL-6 level showed a marked increase in the intestinal manipulation group administered the vehicle (V: 0.5% methylcellulose) alone, but this increase was suppressed by preoperative CT administrations (oral administration once a day for 5 days including the day of surgery), significantly at 70 mg/kg. The GSH levels in the intestine and Peyer’s patches were significantly reduced by intestinal manipulation, but these decreases were significantly inhibited by the preoperative oral CT administration. Moreover, on linear regression analysis of the GSH levels in the intestine and Peyer’s patches and blood IL-6 level, the GSH levels in the intestine and Peyer’s patches both showed significant negative correlations with the blood IL-6 level. From these results, preoperative oral CT administration is considered to suppress the decrease in the intestinal GSH level and increase in the blood IL-6 level associated with intestinal manipulation, i.e., control excessive postoperative inflammatory reactions. Next, to clarify whether or not the suppression of excessive inflammatory reactions by CT leads to early postoperative recovery, behavioral analysis was carried out. While the spontaneous activity level recovered rapidly from Days 1 to 4 after surgery in the sham operation group, little recovery was noted in the manipulation (V) group, and the spontaneous activity level was significantly lower than in the sham operation group on Days 1 to 4. In the manipulation (CT) group, however, the spontaneous activity level recovered gradually from Days 1 to 4, and it was significantly higher than in the manipulation (V) group on Day 4. Regarding the changes in the body weight and food intake from Days 1 to 4, both were significantly lower in the manipulation (V) group than in the sham operation group, but were significantly higher in the manipulation (CT) group than in the manipulation (V) group. From these results in the mouse surgery model, preoperative CT administration is considered to suppress excessive inflammatory reactions and promote postoperative recovery by preventing the decrease in the intestinal GSH level associated with surgical stress (Shibakusa et al. 2012).

#### Postoperative recovery-promoting effect in stomach cancer patients after distal gastrectomy

On the basis of the data obtained in the mouse surgery model, we then carried out a clinical study of stomach cancer patients after distal gastrectomy at Sendai City Medical Center. Forty-three patients aged 75 years or younger underwent elective surgery were randomized to placebo (P) and CT groups. They were given the test food for 10 days from 4 days before surgery, and the resting energy expenditure (REE), blood granulocyte and lymphocyte counts, CRP and IL-6 levels, and body temperature were measured serially. Eventually, 10 patients were excluded due to the retraction of consent and intraoperative complications, and 18 patients in the P group and 15 in the CT group were evaluated. First, the IL-6 level, a blood inflammation marker, showed a peak immediately after surgery and decreased thereafter, but it decreased more rapidly in the CT group and became significantly lower compared with the P group on Day 4 after surgery. Similarly, the CRP level, another blood inflammation marker, showed a peak on Day 1 after surgery and decreased thereafter, but was normalized earlier in the CT group similarly to the IL-6 level, being significantly lower than in the P group on Day 7 after surgery. Also, as mentioned above, CT intake suppressed the increase in granulocytes and decrease in lymphocytes induced by intense exercise, which is acute stress, in athletes (Murakami et al. 2009;Murakami et al. 2010). Therefore, we examined changes in the granulocyte count after gastrectomy, which is also acute stress, compared with the preoperative value. It was increased on Day 1 after surgery and decreased gradually thereafter in both the P and CT groups, but the decrease was faster in the CT group, and became significantly lower than in the P group on Day 4 after surgery. The lymphocyte count was lowest on Day 4 after surgery and recovered thereafter, but no significant difference was noted between the 2 groups. Since the body temperature increases with an inflammatory reaction, we also analyzed postoperative changes in the body temperature as differences compared with the preoperative level. As a result, the temperature was 2.5°C or more higher on the day of surgery compared with the preoperative value and decreased thereafter, but it decreased faster in the CT group than in the P group, and became significantly lower than in the P group on Day 5 after surgery. As there are reports that inflammatory reactions and the REE increased after surgery (Kotani et al. 1996) and that the REE after burn injury, which is an acute stress similar to surgery and intense exercise, was reduced by nutritional therapy with an anti-inflammatory effect (early enteral nutrition) (Mochizuki et al. 1984), we also analyzed the effects of CT administration on the postoperative REE. In the P group, the REE increased to 1.15 times the preoperative level on Day 1 after surgery and decreased thereafter, but had not decreased to the preoperative level even on Day 7 and eventually recovered on Day 14. In the CT group, the REE level did not show an increase as observed in the P group on Day 1 after surgery, when it was significantly lower than in the P group, and remained lower thereafter. From these results, the perioperative administration of CT is considered to accelerate the recovery of the granulocyte count, IL-6, and CRP levels from the postoperative increase and to prevent the postoperative increase in the REE. Therefore, CT administration is considered to promote the postoperative recovery of gastrectomized patients (Miyachi et al. 2013). In the series of trials on intense exercise and surgery mentioned above, we observed the decreasing effects of CT intake on inflammatory markers and immunosuppression, not only in athletes after intense exercise but also in surgery patients. Cystine is a nonessential amino acid. In a clinical trial targeting surgery patients, cystine became essential during the perioperative period (Dale et al. 1977). In addition, the synthetic pathway from methionine to cysteine remains inhibited in rats under surgical stress (Vina et al. 1992). These reports support a role for cystine in suppressing the decrease in GSH during surgical stress. In addition, theanine may act as a glutamate donor in vivo and enhance GSH synthesis via cystine treatment in cells (Rimaniol et al. 2001). Based on these reports and our findings, preoperative oral CT administration has a useful anti-inflammatory and immunosuppressive effect.

## Conclusion and perspectives

**Figure 2:**
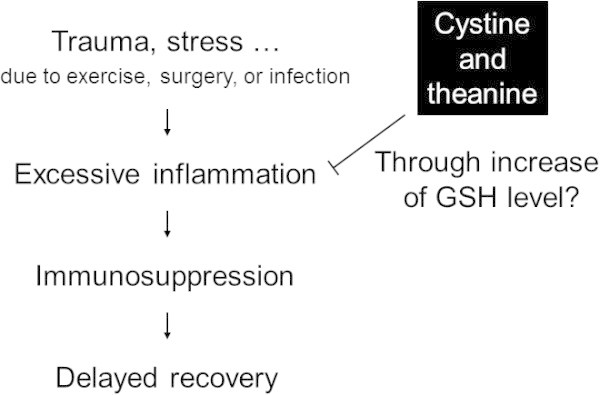
**Scheme of enhanced recovery due to cystine and theanine after the indicated trauma.** Trauma/stress due to exercise, surgery, or infection induces excessive inflammation and immunosuppression. It also delays recovery after trauma. Cystine and theanine suppress the decrease in the GSH level due to trauma, which may inhibit excessive inflammation and immunosuppression. As a result, cystine and theanine enhance recovery after trauma.

As mentioned in this review, CT were shown by clinical studies to not only enhance the antibody-producing ability on infection but also suppress excessive inflammatory reactions induced by intense exercise and surgery. Infection as well as intense exercise and surgery can be regarded as invasive stress. From this viewpoint, CT intake is considered to enhance the antibody-producing ability, control excessive inflammatory reactions, and, in consequence, promote early recovery by inhibiting the decrease in GSH due to invasive stress (Figure 2). However, to prove the hypothesis, more studies are needed that investigate the correlation between GSH levels and immune response to CT intake in mice and humans. While more detailed analysis of the action mechanism is necessary, CT with such effects are expected to be used, for example, as: (1) an oral adjuvant food on the vaccination of older people with reduced immunological functions, (2) a supplement for those who wish to maintain their physical condition throughout the year, (3) a conditioning food for expert and amateur athletes undergoing intensive training, and (4) a medical food to promote postoperative recovery for those expected to undergo surgery (Fearon et al. 2005;Wilmore and Kehlet 2001).

## Abbreviations

APCs: Antigen-presenting cells
CRP: C-reactive protein
CT: Cystine and theanine
GSH: Glutathione
HI: Hemagglutination inhibition
Ig: Immunoglobulin
IL: Interleukin
Mϕ: Macrophage
NK: Natural killer
P: Placebo
REE: Resting energy expenditure
V: Vehicle.

## References

[CR1] Asatoor AM (1966). Tea as a source of urinary ethylamine. Nature.

[CR2] Biffl WL, Moore EE, Moore FA, Peterson VM (1996). Interleukin-6 in the injured patient. Marker of injury or mediator of inflammation?. Ann Surg.

[CR3] Bukowski JF, Morita CT, Brenner MB (1999). Human gamma delta T cells recognize alkylamines derived from microbes, edible plants, and tea: implications for innate immunity. Immunity.

[CR4] Dale G, Young G, Latner AL, Goode A, Tweedle D, Johnston ID (1977). The effect of surgical operation on venous plasma free amino acids. Surgery.

[CR5] Droge W, Holm E (1997). Role of cysteine and glutathione in HIV infection and other diseases associated with muscle wasting and immunological dysfunction. FASEB J.

[CR6] Fearon KC, Ljungqvist O, Von Meyenfeldt M, Revhaug A, Dejong CH, Lassen K, Nygren J, Hausel J, Soop M, Andersen J, Kehlet H (2005). Enhanced recovery after surgery: a consensus review of clinical care for patients undergoing colonic resection. Clin Nutr.

[CR7] Gleeson M, Pyne DB (2000). Special feature for the Olympics: effects of exercise on the immune system: exercise effects on mucosal immunity. Immunol Cell Biol.

[CR8] Grimble RF (2006). The effects of sulfur amino acid intake on immune function in humans. J Nutr.

[CR9] Hara M, Tanaka K, Hirota Y (2005). Immune response to influenza vaccine in healthy adults and the elderly: association with nutritional status. Vaccine.

[CR10] Hayden FG, Osterhaus AD, Treanor JJ, Fleming DM, Aoki FY, Nicholson KG, Bohnen AM, Hirst HM, Keene O, Wightman K (1997). Efficacy and safety of the neuraminidase inhibitor zanamivir in the treatment of influenzavirus infections. GG167 Influenza Study Group. N Engl J Med.

[CR11] Hayden FG, Atmar RL, Schilling M, Johnson C, Poretz D, Paar D, Huson L, Ward P, Mills RG (1999). Use of the selective oral neuraminidase inhibitor oseltamivir to prevent influenza. N Engl J Med.

[CR12] Heikkinen T, Jarvinen A (2003). The common cold. Lancet.

[CR13] Hiki N, Shimizu N, Yamaguchi H, Imamura K, Kami K, Kubota K, Kaminishi M (2006). Manipulation of the small intestine as a cause of the increased inflammatory response after open compared with laparoscopic surgery. Br J Surg.

[CR14] Jefferson T, Rivetti D, Rivetti A, Rudin M, Di Pietrantonj C, Demicheli V (2005). Efficacy and effectiveness of influenza vaccines in elderly people: a systematic review. Lancet.

[CR15] Kalff JC, Schraut WH, Simmons RL, Bauer AJ (1998). Surgical manipulation of the gut elicits an intestinal muscularis inflammatory response resulting in postsurgical ileus. Ann Surg.

[CR16] Kawada S, Kobayashi K, Ohtani M, Fukusaki C (2010). Cystine and theanine supplementation restores high-intensity resistance exercise-induced attenuation of natural killer cell activity in well-trained men. J Strength Cond Res.

[CR17] Kotani G, Usami M, Kasahara H, Saitoh Y (1996). The relationship of IL-6 to hormonal mediators, fuel utilization, and systemic hypermetabolism after surgical trauma. Kobe J Med Sci.

[CR18] Kurihara S, Shibahara S, Arisaka H, Akiyama Y (2007). Enhancement of antigen-specific immunoglobulin G production in mice by co-administration of L-cystine and L-theanine. J Vet Med Sci.

[CR19] Kurihara S, Hiraoka T, Akutsu M, Sukegawa E, Bannai M, Shibahara S (2010). Effects of (L)-cystine and (L)-theanine supplementation on the common cold: a randomized, double-blind, and placebo-controlled trial. J Amino Acids.

[CR20] Luo JL, Hammarqvist F, Andersson K, Wernerman J (1996). Skeletal muscle glutathione after surgical trauma. Ann Surg.

[CR21] MacKinnon LT (2000). Special feature for the Olympics: effects of exercise on the immune system: overtraining effects on immunity and performance in athletes. Immunol Cell Biol.

[CR22] Margonis K, Fatouros IG, Jamurtas AZ, Nikolaidis MG, Douroudos I, Chatzinikolaou A, Mitrakou A, Mastorakos G, Papassotiriou I, Taxildaris K, Kouretas D (2007). Oxidative stress biomarkers responses to physical overtraining: implications for diagnosis. Free Radic Biol Med.

[CR23] McElhaney JE, Beattie BL, Devine R, Grynoch R, Toth EL, Bleackley RC (1990). Age-related decline in interleukin 2 production in response to influenza vaccine. J Am Geriatr Soc.

[CR24] Miyachi T, Tsuchiya T, Oyama A, Tsuchiya T, Abe N, Sato A, Chiba Y, Kurihara S, Shibakusa T, Mikami T (2013). Perioperative oral administration of cystine and theanine enhances recovery after distal gastrectomy: a prospective randomized trial. JPEN J Parenter Enteral Nutr.

[CR25] Miyagawa K, Hayashi Y, Kurihara S, Maeda A (2008). Co-administration of l-cystine and L-theanine enhances efficacy of influenza vaccination in elderly persons: nutritional status-dependent immunogenicity. Geriatr Gerontol Int.

[CR26] Mochizuki H, Trocki O, Dominioni L, Brackett KA, Joffe SN, Alexander JW (1984). Mechanism of prevention of postburn hypermetabolism and catabolism by early enteral feeding. Ann Surg.

[CR27] Mullooly JP, Bennett MD, Hornbrook MC, Barker WH, Williams WW, Patriarca PA, Rhodes PH (1994). Influenza vaccination programs for elderly persons: cost-effectiveness in a health maintenance organization. Ann Intern Med.

[CR28] Murakami S, Kurihara S, Koikawa N, Nakamura A, Aoki K, Yosigi H, Sawaki K, Ohtani M (2009). Effects of oral supplementation with cystine and theanine on the immune function of athletes in endurance exercise: randomized, double-blind, placebo-controlled trial. Biosci Biotechnol Biochem.

[CR29] Murakami S, Kurihara S, Titchenal CA, Ohtani M (2010). Suppression of exercise-induced neutrophilia and lymphopenia in athletes by cystine/theanine intake: a randomized, double-blind, placebo-controlled trial. J Int Soc Sports Nutr.

[CR30] Nieman DC (1997). Risk of upper respiratory tract infection in athletes: an epidemiologic and immunologic perspective. J Athl Train.

[CR31] Nordin J, Mullooly J, Poblete S, Strikas R, Petrucci R, Wei F, Rush B, Safirstein B, Wheeler D, Nichol KL (2001). Influenza vaccine effectiveness in preventing hospitalizations and deaths in persons 65 years or older in Minnesota, New York, and Oregon: data from 3 health plans. J Infect Dis.

[CR32] Remarque EJ, Cools HJ, Boere TJ, van der Klis RJ, Masurel N, Ligthart GJ (1996). Functional disability and antibody response to influenza vaccine in elderly patients in a Dutch nursing home. BMJ.

[CR33] Rimaniol AC, Mialocq P, Clayette P, Dormont D, Gras G (2001). Role of glutamate transporters in the regulation of glutathione levels in human macrophages. Am J Physiol Cell Physiol.

[CR34] Scheid L, Ellinger S, Alteheld B, Herholz H, Ellinger J, Henn T, Helfrich HP, Stehle P (2012). Kinetics of L-theanine uptake and metabolism in healthy participants are comparable after ingestion of L-theanine via capsules and green tea. J Nutr.

[CR35] Shibakusa T, Mikami T, Kurihara S, Chiba Y, Tsuchiya T, Miyachi T, Oyama A, Tanaka KA, Koyama N (2012). Enhancement of postoperative recovery by preoperative oral co-administration of the amino acids, cystine and theanine, in a mouse surgical model. Clin Nutr.

[CR36] Smith L (2003). Overtraining, excessive exercise, and altered immunity: is this a T helper-1 versus T helper-2 lymphocyte response?. Sports Med.

[CR37] Suzuki K, Yamada M, Kurakake S, Okamura N, Yamaya K, Liu Q, Kudoh S, Kowatari K, Nakaji S, Sugawara K (2000). Circulating cytokines and hormones with immunosuppressive but neutrophil-priming potentials rise after endurance exercise in humans. Eur J Appl Physiol.

[CR38] Takagi Y, Kurihara S, Higashi N, Morikawa S, Kase T, Maeda A, Arisaka H, Shibahara S, Akiyama Y (2010). Combined administration of (L)-cystine and (L)-theanine enhances immune functions and protects against influenza virus infection in aged mice. J Vet Med Sci.

[CR39] Vina J, Gimenez A, Puertes IR, Gasco E, Vina JR (1992). Impairment of cysteine synthesis from methionine in rats exposed to surgical stress. Br J Nutr.

[CR40] Vu T, Farish S, Jenkins M, Kelly H (2002). A meta-analysis of effectiveness of influenza vaccine in persons aged 65 years and over living in the community. Vaccine.

[CR41] Wilmore DW, Kehlet H (2001). Management of patients in fast track surgery. BMJ.

[CR42] Windmueller HG, Spaeth AE (1975). Intestinal metabolism of glutamine and glutamate from the lumen as compared to glutamine from blood. Arch Biochem Biophys.

